# Polarization Doping in a GaN-InN System—Ab Initio Simulation

**DOI:** 10.3390/ma16031227

**Published:** 2023-01-31

**Authors:** Ashfaq Ahmad, Pawel Strak, Pawel Kempisty, Konrad Sakowski, Jacek Piechota, Yoshihiro Kangawa, Izabella Grzegory, Michal Leszczynski, Zbigniew R. Zytkiewicz, Grzegorz Muziol, Eva Monroy, Agata Kaminska, Stanislaw Krukowski

**Affiliations:** 1 Institute of High Pressure Physics, Polish Academy of Sciences, Sokolowska 29/37, 01-142 Warsaw, Poland; 2 Research Institute for Applied Mechanics, Kyushu University, Fukuoka 816-8580, Japan; 3 Institute of Applied Mathematics and Mechanics, University of Warsaw, 02-097 Warsaw, Poland; 4 Institute of Physics, Polish Academy of Sciences, Aleja Lotnikow 32/46, 02-668 Warsaw, Poland; 5 University Grenoble-Alpes, CEA, Grenoble INP, IRIG, PHELIQS, 17 av. des Martyrs, 38000 Grenoble, France; 6 Faculty of Mathematics and Natural Sciences, School of Exact Sciences, Cardinal Stefan Wyszynski University, Dewajtis 5, 01-815 Warsaw, Poland

**Keywords:** polarization doping, nitrides, InGaN, graded layer

## Abstract

Polarization doping in a GaN-InN system with a graded composition layer was studied using ab initio simulations. The electric charge volume density in the graded concentration part was determined by spatial potential dependence. The emerging graded polarization charge was determined to show that it could be obtained from a polarization difference and the concentration slope. It was shown that the GaN-InN polarization difference is changed by piezoelectric effects. The polarization difference is in agreement with the earlier obtained data despite the relatively narrow bandgap for the simulated system. The hole generation may be applied in the design of blue and green laser and light-emitting diodes.

## 1. Introduction

Wurtzite crystalline symmetry permits emergence of a macroscopic vectorial quantity known as polarization [1]. Macroscopically, polarization occurs due to relative translation of the center of the negative electrons with respect to the position of positive atomic nucleus, i.e., creation of electric dipole density [1]. This is associated directly with the iconicity of metal–nonmetal bonding. Nitride semiconductors such as GaN, AlN and InN have different fractions of the covalent and ionic components in their bonding. Therefore, the chemically induced charge effect is different, which leads to a difference in their spontaneous polarization. In addition, external strain can downgrade the symmetry of the lattice, which could induce or change the polarization charge [2,3].

Polarization affects physical properties of semiconductor systems by the emergence of the electric fields of various magnitudes and ranges. In large size systems, the large scale electric fields are negligible due to screening which is described in Debye-Hückel or Thomas-Fermi approximation [3,4,5]. A much stronger influence of polarization-related electric fields is observed in nanometer size systems. A glaring, positive example is the localization of electrons in the GaN-based field-effect transistor (FET) [6]. For III-nitride multi-quantum well (MQWs) laser diodes (LDs) and light-emitting diode (LED) performance, these fields are highly detrimental. The electron–hole wavefunction overlap and accordingly the radiative recombination dipole strength is reduced by the so called quantum-confined Stark effect (QCSE) [7,8,9]. The efforts to avoid the fields in the devices using nonpolar MQWs were not successful due to the poor material quality in such structures [10].

In devices containing heterostructures, polarization difference entails a sheet charge [11,12] and a surface dipole layer [12] at the heterointerfaces. The sheet charge is equivalent to the electric field difference across the interface. The phenomenon is well understood; however the magnitude is still under debate [13,14,15]. At the dipole layer region, the electric potential jumps between both sides of the interface. The effect was identified a long time ago, but it is still disputed [16].

Chemically induced electric phenomena are not limited to sharp interfaces. The transition between two chemically different substances may be also more or less diffuse. Predictably, the diffuse interface generates the extended charge density [17,18,19,20,21,22,23]. The nitride alloys concentration change leads to the polarization difference and creation of a bulk charge density, inducing a phenomenon known as polarization doping [18,19,20,21,22,23]. Since the polarization is proportional to metal concentration, linearly graded materials have a uniform bulk charge density. In insulating systems, without screening the potential profile, it can be used to determine the polarization-related charge density via the Poisson equation. The method was successfully used to determine the polarization density constant for the Al-Ga-N system in [23]. In addition, the mechanism for the generation of the mobile charge was described and used to design the UV laser diode (LD).

The development of blue nitride-based lasers was one of the most spectacular achievements, in terms of scientific achievements, development of technology and contribution to society [24,25]. Unfortunately, the extension of the emission spectrum towards UV [26,27] and green [28,29] light emission encounters considerable difficulties. The problem of p-type doping is one of the roadblocks towards high power green UV lasers which have many promising applications. The best possible remedy is to use Mg and polarization doping simultaneously [30]. In this work, we will use the methods developed in [23] for the Ga-In-N system to obtain polarization-doping constants, verify the creation of mobile charge and its application to device design.

## 2. Methods

Ab initio simulations reported in this paper employed the density functional theory (DFT) package SIESTA. SIESTA uses numeric atomic orbital series to solve Kohn–Sham equations [23,24]. The following atomic orbitals were used: for In—3s: DZ (double zeta), 3p: TZ (triple zeta), 3d: DZ; N—2s: DZ, 2p: TZ, 3d: SZ (single zeta) and for Ga- 4s: DZ (double zeta), 4p: TZ (triple zeta), 3d: DZ. The pseudopotentials approximation was used for all atoms which allowed us to increase the number of atoms and consequently, the size of supercell used. The pseudopotentials were generated by the authors using the ATOM program designed for all-electron calculations. In SIESTA the norm-conserving Troullier–Martins pseudopotentials, in the Kleinmann–Bylander factorized form are used [31,32,33,34]. The calculations were performed within Generalized Gradient Approximation (GGA) with the PBEsol modified version of the original Perdew, Burke and Ernzerh of the exchange–correlation functional [35,36].

The lattice parameters of bulk indium nitride, calculated for a periodic infinite crystal, are $a_{InN}^{DFT}=3.525\;Å$ and $c_{InN}^{DFT}=5.716\;Å$. They are in sufficient agreement with the experimental data for wurtzite bulk InN obtained from X-ray diffraction measurements: $a_{InN}^{exp}=3.537\;Å$ and $c_{InN}^{exp}=5.703\;Å$ [37]. Ab initio calculations of GaN gave $a_{GaN}^{DFT}=3.198\;Å$ and $c_{GaN}^{DFT}=5.199\;Å$, again showing agreement with the X-ray data $a_{GaN}^{exp}=3.1890\;Å$ and $c_{GaN}^{exp}=5.1864\;Å$ [38]. PBEsol approximation provides an erroneous result for band structure in which the gap is closed. Therefore, the band diagrams were plotted from calculations using Ferreira et al. GGA-1/2 approximation [39,40]. It provides effective masses, bandgap (BG) energies, and more generally, the band structures are in agreement with experimental data [40]. The GGA-1/2 approximation calculations were used for the positions of atoms and a periodic cell parameter relaxed to equilibrium in GGA-PBEsol approximation. All atom positions were changed to reduce the single atom forces at a level below 0.005 eV/Å. The bandgap of the InN crystal was: $E_{g}^{DFT}\left(InN\right)=0.75\;eV$. The bandgap of InN was the subject of initial controversy. Finally, the experimental data of several papers established InN gap value as ($E_{g}^{exp}\left(InN\right)=0.65\;eV$) [41,42,43]. The ab initio gap value of GaN was $E_{g}^{DFT}\left(GaN\right)=3.51\;eV$. This could be compared to the well-established experimental GaN bandgap $E_{g}^{exp}\left(GaN\right)=3.47eV$ [44,45]. A convergence criterion for a self-consistent field (SCF) loop termination was that the maximum difference between the output and the input for any element of the density matrix had to be below 10^−4^.

## 3. Results

**Figure 1:**
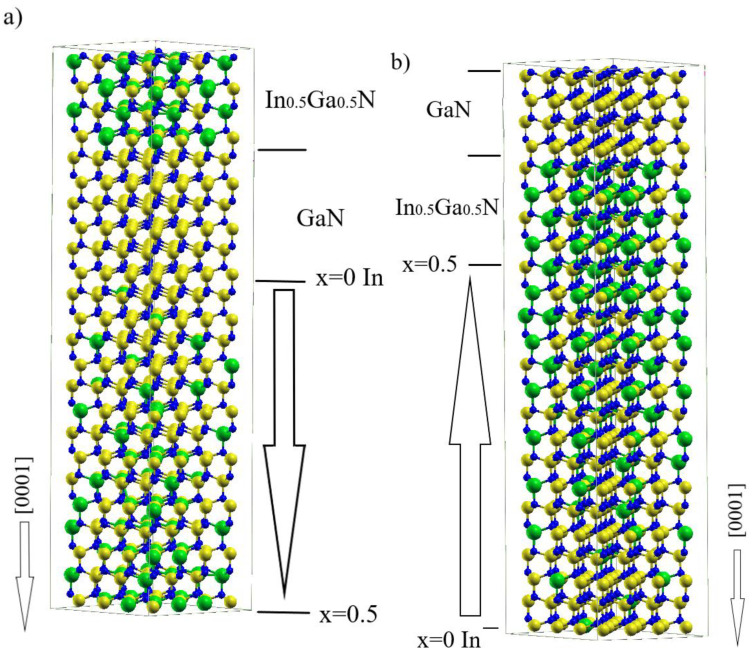
(**a**) In_x_Ga_1−x_N/GaN/In_0.5_Ga_0.5_N and (**b**) In_x_Ga_1−x_N/In_0.5_Ga_0.5_N/GaN supercells that were subject to ab initio calculations. The Ga_x_In_1−x_N layer is graded linearly, with (**a**) increasing and (**b**) decreasing In concentration towards the bottom of the ([0001] direction). Ga, In and N atoms are represented by yellow, green and blue balls, respectively.

Ab initio calculations were used to simulate the In-Ga-N supercell composed of the three chemically different regions, arranged along the c-axis, i.e., along [0001] direction: (i) uniform GaN layer, (ii) uniform In_0.5_G_0.5_N layer, (iii) graded In_x_Ga_1−x_N layer (0.0≤x≤0.5). This is a direct implementation of the calculation method successfully used in determination of polarization doping in an AlN-GaN system [23]. As in the standard supercell DFT calculations, the periodic boundary conditions are imposed on all boundaries of the supercell. The atomic arrangement in the supercell is (Figure 1):

(i) Four Ga-N double atomic layers (metal and nonmetal layers);
(ii) Four In_0.5_Ga_0.5_N double atomic layers (metal and nonmetal layers);
(iii) Sixteen linearly graded Ga-In-N double atomic layers, with Ga content increasing or decreasing along [0001] direction (metal layers composed of Ga and In, nonmetal layer composed of N).

**Figure 2:**
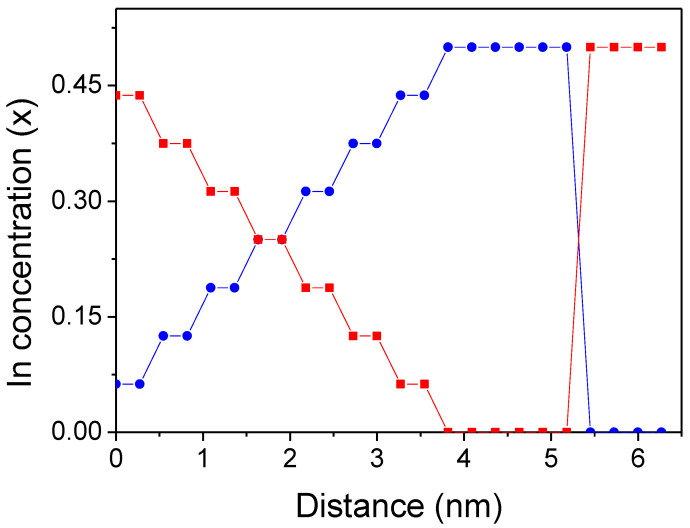
Indium concentration (x) along the [000-1] axis in the structures presented in Figure 1. The zero coordinate is set at the bottom of the cell. The red circles and the blue squares represent indium concentration in the lattice plotted in Figure 1a,b, respectively. The line is for guiding the eye only.

**Figure 3:**
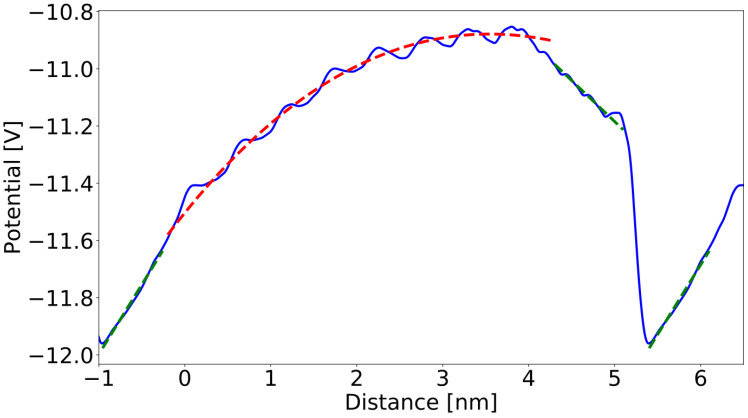
The averaged potential profile obtained from ab initio calculations of the structures in Figure 1a. The zero coordinate value is set at the bottom of the cell. Solid blue lines represent averaged potential profiles, red dashed lines—parabolic approximation in the graded regions; green dashed lines—linear approximation in the uniform regions.

In the ternary alloy section, the metal atoms are distributed randomly within each layer independently. The number of In atoms in the neighboring layers changes every two layers. Thus, the number of the In and Ga atoms is set in the layer so that average concentration is changed along the *z*-axis linearly (Figure 2). The lattice positions of In and Ga atoms within the layer are generated based on a random number of generators employed in many Monte Carlo simulations. Therefore, the selection enforces uniform sampling in the lattice sites, i.e., the method not prone to systematic errors, generating a relatively high level of numeric noise. The potential profiles presented in Figure 3 confirm that the random factor is not important. This remarkable effect results from a long range of Coulomb interactions that smooth out the potential profile. In the effect, the potential profile does not show any considerable local variations related to InGaN configuration. The selected positions of In and Ga atoms in the neighboring layers are uncorrelated. In addition, several configurations were used to obtain the averaged quantities.

The averaged composition of the atomic layers is presented in Figure 2. The graded region consist of eight unit cells in which the In concentration changed from 0 to 0.5. Therefore, the average concentration change across the whole graded layer, i.e., at the approximate distance of 8c =4.3612 nm was Δx=0.5. The resulting concentration slope is $sc=\frac{dx}{dz}=0.115\;{\mathrm{nm}}^{-1}$.

The polarization is expected to vary linearly with the In and Ga concentrations. The polarization change should give a bulk charge density $\rho \left(\vec{r}\right)$, which is related to the electrical potential $\varphi \left(\vec{r}\right)$ in accordance with Poisson equation:

(1) $$\nabla ∙\left(\varepsilon_{b}\left(\vec{r}\right)\nabla \varphi \left(\vec{r}\right)\right)=\frac{-\rho_{pd}\left(\vec{r}\right)}{\varepsilon_{o}}$$

where $\varepsilon_{b}$ is the static dielectric constant of the semiconductor and $\varepsilon_{o}$ dielectric permittivity. The dielectric constants of InN and GaN are different therefore, the Vegard law is used for graded region $\varepsilon_{b}\left(x\right)=x\;\varepsilon_{b-InN}+\left(1-x\right)\;\varepsilon_{b-GaN}$, where $\varepsilon_{b-InN}=14.61$ and $\varepsilon_{b-GaN}=10.28$ [46]. Using this approximation, the variation of the dielectric constant along z coordinate in the graded region can be represented as $\varepsilon_{b}\left(z\right)=q_{1}+q_{2}z$, where these parameters are:

(2a) $$q_{1}=\varepsilon_{b-GaN},\;q_{2}=\left(\varepsilon_{b-GaN}-\varepsilon_{b-InN}\right)\;sc$$

(2b) $$q_{1}=\left(\varepsilon_{b-GaN}+\varepsilon_{b-InN}\right)/2\;,\;q_{2}=\left(\varepsilon_{b-GaN}-\varepsilon_{b-InN}\right)\;sc$$

The electric potential profile was obtained by exact analytical solution of Equations (1) and (2) within the graded region to obtain

(3) $$\varphi \left(z\right)\;=\;a_{0}+a_{1}\log\left(q_{1}+q_{2}z\right)-\frac{\rho_{pd}}{\varepsilon_{o}q_{2}}z\;=\;a_{0}+a_{1}\log\left(q_{1}+q_{2}z\right)+a_{2}z$$

where constants *a*_0_ and *a*_1_ depend on the boundary conditions, and a_2_ depends on polarization charge ρ_pd_, electric constant *ε*_0_ and parameter *q*_1_. In this case, boundary conditions are not used, as constants a_0_, a_1_ and a_2_ are fitted to the potential obtained from the profile that is averaged in the plane perpendicular to c-axis, derived from ab initio calculations. The plane averaged potential is highly oscillatory due to maxima from atomic core charges. The basic procedure is smoothing by adjacent averaging over the c lattice parameter period. Still, the potential oscillations cannot be completely averaged out due to the change of the lattice constant in the graded region.

On the other hand, in non-graded regions, parameter *ε_b_* is constant and we obtain a linear potential, as the averaged bulk charge is zero. The obtained potential profiles corresponding to the supercell presented in Figure 1a are shown in Figure 3.

The segments of the profile obtained in the uniform GaN, uniform In_0.5_Ga_0.5_N and linearly graded In_x_Ga_1−x_N have distinctively different space dependence. The sections with uniform concentration are linear, whereas the potential profile within the linearly graded alloy are parabolic, typical for the systems having uniform bulk charge density. In addition, at the In_0.5_Ga_0.5_N/GaN interface, a potential jump is observed, due to the emergence of a dipole layer. The two different averaging procedures provided slightly different profiles: more or less oscillatory in the uniform and graded regions and the different potential jump at the In_0.5_Ga_0.5_N/GaN interface.

As it was shown, the potential profile in the graded region closely follows the solution (3) confirming the assumption of uniform charge density in this region. The following parameters were obtained: $a_{0}=-134\;V$, $a_{1}=52.7\;V$ and $a_{2}=-2.173\frac{V}{\mathrm{nm}}$. The bulk charge density derived via Equations (2a) and (3) is: $\rho_{\mathrm{pd}}=9.55\times 10^{6}\frac{C}{m^{3}}$ (i.e., 5.96 × 10^19^ cm^−3^). From the geometric data, the thickness of the graded region is $d_{\mathrm{grad}}≅4.3612\;\mathrm{nm}$. Thus, the obtained surface sheet charge density is $\rho_{surf}=4.165\times 10^{-2}\frac{C}{m^{2}}$.

In the uniform regions, the potential profiles are linear, confirming the absence of the bulk charge density. From the linear approximation, the electric field can be derived. The following field values were obtained: ${\vec{E}}_{GaN}\left(a\right)=-\nabla \varphi =0.287\;V/\mathrm{nm}=2.87\times 10^{-2}V/Å$ and ${\vec{E}}_{GaInN}\left(a\right)=-\nabla \varphi =-0.487\;V/\mathrm{nm}=-4.87\times 10^{-2}\;V/Å$. The sheet charge density at the interfaces obtained via Gauss law ($\vec{n}$—unit normal vector, perpendicular to the interface):

(4) $$\rho_{s}=\varepsilon_{o}\left[\left(\frac{\varepsilon_{GaN}+\varepsilon_{InN}}{2}\right){\vec{E}}_{GaInN}-\varepsilon_{GaN}{\vec{E}}_{GaN}\right]\;\vec{n}=\left({\vec{P}}_{GaInN}-{\vec{P}}_{GaN}\right)\;\vec{n}$$

is $\rho_{s}=-7.59\times 10^{-2}C/m^{2}$. This has to be compared with the estimate obtained above by integration of bulk charge over the thickness of the graded region which was $\rho_{surf}=4.165\times 10^{-2}C/m^{2}$. The difference is due to interfacial charges present on the interfaces between the graded layer and neighboring layers, which do not contribute to the spatial polarization charge.

**Figure 4:**
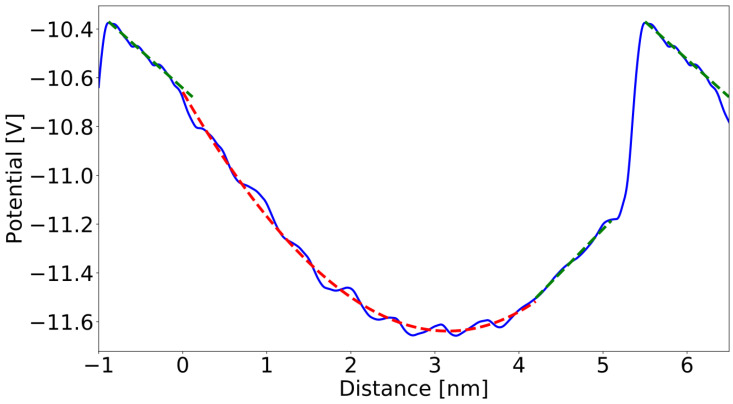
The averaged electric potential profile obtained from ab initio calculations of the structures in Figure 1b. The zero distance is set at the bottom of the cell. Solid blue lines represent averaged potential profiles, red dashed lines—parabolic approximation in the graded regions; green dashed lines—linear approximation in the uniform regions.

Similar analysis could be made for the case presented in Figure 1b. The obtained potential profiles, corresponding to the supercell presented in Figure 1b are shown in Figure 4. From the fit to Formula (3), the following parameters were obtained: $a_{0}=250\;V$, $a_{1}=-103\;V/\mathrm{nm}$ and $a_{2}=-4.73\;V/{\mathrm{nm}}^{2}$. The charge density derived via Equation (3) is: $\rho_{pd}=2.08\times 10^{7}C/m^{3}$ (i.e., 1.3 × 10^20^ cm^−3^). From the geometric data, the thickness of graded region is $d_{grad}≅4.3612\;\mathrm{nm}$. Thus, the obtained surface sheet charge density is $\rho_{surf}=-9.065\times 10^{-2}\frac{C}{m^{2}}$.

In the uniform regions, the potential profiles are linear, which confirms the absence of the bulk charge density. The following field values were obtained from linear approximations: ${\vec{E}}_{GaN}\left(a\right)=-\nabla \varphi =-0.353V/\mathrm{nm}=-3.53\times 10^{-2}V/Å$ and ${\vec{E}}_{GaInN}\left(a\right)=-\nabla \varphi =0.309V/\mathrm{nm}=3.09\times 10^{-2}V/Å$. The surface charge density at the interfaces obtained from Gauss law (Equation (4)) is $\rho_{s}=6.62\times 10^{-2}C/m^{2}$. This has to be compared with the estimate obtained above by integration of bulk charge over the thickness of the graded region which was is $\rho_{surf}=-9.07\times 10^{-4}C/m^{2}$. Again, the difference is due to interfacial charges present on the interfaces between the graded layer and neighboring layers.

In summary, the surface density is obtained in case (a) from the bulk density $\rho_{surf}=4.165\times 10^{-2}\frac{C}{m^{2}}$. In the second case, this value is $\rho_{surf}=-9.065\times 10^{-2}\frac{C}{m^{2}}$. The discrepancy between case (a) and (b) may be related to the fact that at the Ga_0.5_In_0.5_N/Ga_x_In_1−x_N system bandgap is small so the mobile band charge may affect the obtained potential profile and the resulting polarization doping bulk charge. Therefore, the polarization doping bulk charge obtained is $\rho_{pd}=9.55\times 10^{6}\frac{C}{m^{3}}$ and $\rho_{pd}=-2.079\times 10^{7}\frac{C}{m^{3}}$ in case (a) and (b) respectively. This could be translated into the elementary charge density (a) $n=5.97\;\times \;10^{19}\;{\mathrm{cm}}^{-3}$ and (b) $p=1.3\;\times \;10^{20}\;{\mathrm{cm}}^{-3}$. From these, the values of polarization doping parameters $Q_{pol}$ could be obtained as:

(5) $$Q_{pol}=\;\frac{\rho_{pd}}{e\;sc}=\frac{\Delta P}{e}$$

From these calculations, the values of polarization doping parameters are: (a) $Q_{pol}=0.52\;\;{\mathrm{nm}}^{-2}$ and (b) $Q_{pol}=-1.13\;\;{\mathrm{nm}}^{-2}$. Thus these values can be used to calculate the InN-GaN polarization difference as: (a) $\Delta P_{GaN-InN}=\;P_{GaN}-\;P_{InN}=\;Q_{pol}\;e=0.083\;\frac{C}{m^{2}}$ and (b) $\Delta P_{GaN-InN}=\;P_{GaN}-\;P_{InN}=\;-Q_{pol}\;e=0.181\;\frac{C}{m^{2}}$. The critical evaluation of the polarization difference was given in [46]. The obtained polarization difference was calculated using the piezoelectric constants from [13,14]. In the calculations, the elastic constants obtained from Mahata et al. [47] have been used. Invoking the mechanical stability rule $\varepsilon_{33}=-2C_{13}\varepsilon_{11}/C_{33}$, the polarization difference may be obtained as a function of a lattice parameter. The data collected in [46] indicate that the polarization difference obtained in [13] changes from $\Delta P_{GaN-InN}=0.09C/m^{2}\;$for a=3.113 nm to $\Delta P_{GaN-InN}=0.10C/m^{2}\;$for a=3.195 nm [13,46]. Ref. [14] provides similar dependence on the lattice parameters but the values are systematically lower $\Delta P_{GaN-InN}=0.07C/m^{2}\;$for a=3.113 nm to $\Delta P_{GaN-InN}=0.09C/m^{2}\;$for a=3.113 nm [14,46]. These values are slightly smaller than those obtained in the present simulations. Nevertheless, basic agreement between the earlier results and present simulations was obtained, proving the validity of the present approach.

**Figure 5:**
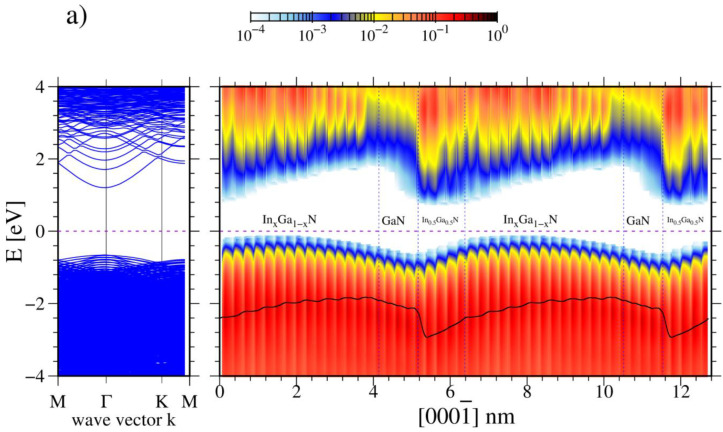

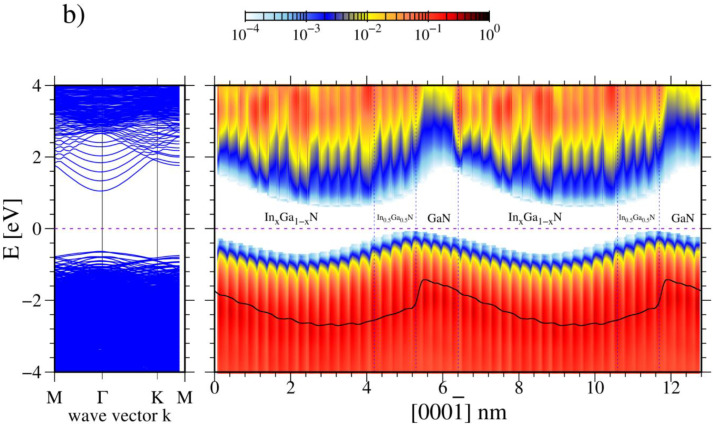
Band profiles of the lattices presented in (**a**,**b**) These diagram show: left panel—energy bands in the momentum space, right panel—energy bands in the coordination space. The color at the top gives the scale of the density of states. The black line that is superimposed on the valence band represents the averaged potential multiplied by the electron charge.

Finally, the In_x_Ga_1−x_N/GaN/In_0.5_Ga_0.5_N and In_x_Ga_1−x_N/In_0.5_Ga_0.5_N/GaN structures described in Figure 1 have band profiles calculated from ab initio data by a projection of band states on the atom row states. This exact procedure was used previously for both AlN/GaN MQWs [16] and surface slab [48] simulations. The obtained results, presented in Figure 5, indicate that the Fermi level is located in the bandgap so the contribution of free carriers is small. In fact, the low InN bandgap leads to a small energy difference between the valence band maximum (VBM) and the Fermi energy in the fraction of the simulated system. It is expected that due to the relatively small InN bandgap ($E_{g}^{DFT}\left(InN\right)=0.75\;eV$) compared to a wide GaN energy gap ($E_{g}^{DFT}\left(GaN\right)=3.51\;\mathrm{eV}$), the smallest VBM-Fermi energy distance should be observed in Ga_0.5_In_0.5_N layer. It is not due to the combination of the energy change and the induced electric fields in case (a) that causes the highest VBM energy and is located in the graded In_x_Ga_1−x_N layer interior about 1 nm from the In_0.5_Ga_0.5_N/In_x_Ga_1−x_N interface. The estimated energy difference is about 0.1 eV; therefore, the screening by the mobile charge may contribute to the potential profile in the graded region. In case (b), the highest VBM energy is located at the In_0.5_Ga_0.5_N/GaN interface, due to the contribution of the polarization induced charge and dipole density. Again, we expect that this contribution may affect the obtained electric field jump at this interface. Therefore, the deviation in polarization difference obtained here and reported in the previous publications may be attributed to these factors [13,14,46].

From the point of view of possible applications, the emergence of the mobile carriers in addition to the immobile charge of the polarization-doping system is crucial, as discussed in [23]. It was found that in the case of Al_x_Ga_1−x_N graded systems, the widening of the uniform part of GaN or In_0.5_Ga_0.5_N leads to penetration of the Fermi level in the conduction and the valence bands, so that the mobile carriers can screen the field. In Al_x_Ga_1−x_N graded systems, the effect was more symmetric, the Fermi level was located at the midgap. In the case of the In_x_Ga_1−x_N graded system, the Fermi level was located in the VBM vicinity. This opens the possibility of relatively easy emergence of holes which are particularly interesting in long wavelength devices, such as green and red laser diodes (LDs) and light-emitting diodes (LEDs). The solution of the p-type doping of In-containing parts of the device may be facilitated by the use of In_x_Ga_1−x_N graded layers [22]. The design of such devices is possible through the use of drift-diffusion-based software [49,50,51,52,53,54]. This software was used in the simulation of nitride devices based on Al_x_Ga_1−x_N graded systems [48]. The effect has immense technological importance as scattering by ionized defects is absent [55,56]. Thus, alloy scattering remains the dominant scattering process limiting carrier mobility [19,55,57]. Recently, polarization doping was successfully used in the design of AlGaN UVB LEDs proving that the concept could be used in nitride optoelectronic devices [30,58]. The present work opens its application in InGaN systems for the design of long wavelength LEDs and LDs.

## 4. Conclusions

Ab initio simulations of the In_x_Ga_1−x_N/In_o.5_Ga_0.5_N/GaN system proved the existence of the bulk polarization-doping immobile charge in the graded In_x_Ga_1−x_N layer. It was shown that the bulk charge can be obtained using the following relation $\rho_{pd}=\Delta P\left(\frac{dx}{dz}\right)$ where the InN-GaN polarization difference is $\Delta P=\Delta P_{GaN-InN}=P_{GaN}-P_{InN}$. The resulting polarization difference is $\Delta P_{GaN-InN}=P_{GaN}-P_{InN}=0.13\;\mp 0.02\;C/m^{2}$. The polarization difference is dependent on the lattice parameter via strong piezoelectric effects. Both these results, i.e., the magnitude and the piezo-dependence, are in accordance with the earlier critical evaluation of the polarization in [46] and the earlier obtained results in [13,14,46].

The obtained results are affected by several factors, characteristic of the investigated systems. InN is a narrow band material which entails a relatively small difference of the VBM and Fermi energy. Therefore, the mobile band charge (holes) may affect the electric potential distribution used to determine the value of the bulk charge density.

The obtained results indicate the existence of the polarization induced charge density in nitride heterostructures, in accordance with the polarization difference. In addition, the potential jumps of the order of 1 V at the interfaces prove the existence of the surface dipole layer at nitride heterointerfaces.

These results could be potentially applied in the p-type polarization doping of the In-rich structures used in green and red LEDs and LDs technology. Therefore, application of such a design may be highly beneficial in the extension of the emitted light range to longer wavelengths.

## Data Availability

The data that support the findings of this study are available from the corresponding author upon reasonable request.
